# 低凝血酶原血症-狼疮抗凝物综合征1例并文献复习

**DOI:** 10.3760/cma.j.cn121090-20241129-00496

**Published:** 2024-12

**Authors:** 泮婧 王, 葳 刘, 婷 孙, 玉华 王, 仁池 杨, 磊 张, 峰 薛

**Affiliations:** 1 中国医学科学院血液病医院（中国医学科学院血液学研究所），血液与健康全国重点实验室，国家血液系统疾病临床医学研究中心，细胞生态海河实验室，天津市血液病基因治疗研究重点实验室，中国医学科学院血液病基因治疗重点实验室，天津 300020 State Key Laboratory of Experimental Hematology, National Clinical Research Center for Blood Diseases, Haihe Laboratory of Cell Ecosystem, Tianjin Key Laboratory of Gene Therapy for Blood Diseases, CAMS Key Laboratory of Gene Therapy for Blood Diseases, Institute of Hematology & Blood Diseases Hospital, Chinese Academy of Medical Sciences & Peking Union Medical College, Tianjin 300020, China; 2 天津医学健康研究院，天津 301600 Tianjin Institutes of Health Science, Tianjin 301600, China; 3 中国医学科学院北京协和医学院群医学及公共卫生学院，北京 100730 School of Population Medicine and Public Health, Chinese Academy of Medical Sciences and Peking Union Medical College, Beijing 100730, China

## Abstract

低凝血酶原血症-狼疮抗凝物综合征（HLAS）是一种罕见的凝血异常疾病，通常以出血为临床表现，特征性凝血酶原活性降低和狼疮抗凝物阳性。但其实验室检查结果复杂，易延误诊疗。本文报道了1例以反复出血为临床表现的11岁HLAS女性患儿。凝血相关检查提示APTT、PT延长，凝血酶原活性减低，APTT纠正试验提示即刻抑制型抗体存在，狼疮抗凝物阳性，抗心磷脂抗体、抗β_2_糖蛋白1（β2GP1）抗体、抗磷脂酰丝氨酸/凝血酶原（aPS/PT）抗体阳性，因此诊断为HLAS，患者同时诊断系统性红斑狼疮，予激素治疗后凝血酶原活性恢复正常。

## Abstract (en)

The Hypoprothrombinemia-Lupus Anticoagulant Syndrome（HLAS）is a rare coagulation disorder, typically presenting with bleeding manifestations. It is characterized by decreased prothrombin activity and the presence of lupus anticoagulant, but laboratory findings are complex, which can delay diagnosis and treatment. This report describes the diagnosis and management of a HLAS patient who was an 11-year-old girl with recurrent bleeding. Coagulation tests revealed prolonged APTT and PT, decreased prothrombin activity, and immediate inhibitory antibodies in the APTT correction test. Lupus anticoagulant, antiphospholipid antibodies, anti-β_2_-glycoprotein 1 antibodies, and phosphatidylserine/prothrombin（aPS/PT）antibodies were positive, leading to a diagnosis of Hypoprothrombinemia-Lupus Anticoagulant Syndrome. The patient was also diagnosed with systemic lupus erythematosus. Following corticosteroid therapy, prothrombin activity returned to normal.

低凝血酶原血症-狼疮抗凝物综合征（HLAS）是一种罕见的凝血异常疾病，其实验室检测要点为凝血酶原活性（FⅡ∶C）降低及狼疮抗凝物（LA）阳性。与LA阳性患者血栓多见的临床表现不同，HLAS患者通常表现为出血，为临床诊断提供了一定挑战。本文报道1例以出血为临床表现的HLAS患者的诊治过程，并进行文献复习。

## 病例资料

患者女，11岁，因“反复鼻衄伴皮肤瘀斑2个月余”就诊。患者就诊前2个月余无明显诱因出现反复鼻衄难止及皮肤瘀斑，多次就诊于当地医院，血常规检查：WBC、PLT正常，HGB自124 g/L逐渐下降至55 g/L。多次查凝血功能：PT 21.1～40.3 s、APTT 59.0～254.6 s；凝血因子活性：FⅡ 7.8％、FⅤ 58.1％、FⅦ 76.8％、FⅧ 22.9％、FⅨ 11.4％、FⅩ 70.3％、FⅪ 16.2％、FⅫ 12.7％；出凝血疾病基因检测未见异常。给予维生素K、新鲜冰冻血浆、凝血酶原复合物（PCC）输注后出血好转但易反复，遂于我院就诊。患者既往无殊。查体：体温36.8 °C、心率111次/min、呼吸21次/min、血压121/83 mmHg（1 mmHg＝0.133 kPa）、发育正常，重度贫血貌，周身散在瘀斑，余未见明显异常。

我院检验及检查：血常规：WBC 5.11×10^9^/L、NEU 2.42×10^9^/L、HGB 58 g/L、PLT 366×10^9^/L、网织红细胞百分比（RET％）6.12、平均红细胞体积（MCV）83.8 fl、平均血红蛋白浓度（MCHC）312 g/L；生化：白蛋白（ALB）34.1 g/L、球蛋白（GLB）38.5 g/L、谷丙转氨酶（ALT）53.2 U/L、谷草转氨酶（AST）54.3 U/L、铁蛋白8.7 ng/ml、血清铁3.44 µmol/L、未饱和铁53.05 µmol/L。尿潜血（+）、尿蛋白（+）、大便隐血（+）。凝血功能：PT 43.5 s、APTT 106.9 s、纤维蛋白原（Fg）1.83 g/L、D-二聚体<0.19 mg/L FEU。凝血因子活性：FⅡ活性明显降低，内源性凝血因子活性检测呈明显稀释现象即活性随稀释度增加而升高，提示受非特异抗体干扰，FⅩⅢ筛选阴性。APTT纠正试验提示即刻抑制型抗体存在。FⅡ抑制物定量（Bethesda法）：0。血管性血友病因子抗原、活性正常。免疫球蛋白定量+风湿3项：IgA 3.12 g/L、IgG 21.7 g/L、IgM 0.78 g/L、C3 0.12 g/L、C4 0.017 g/L、抗链球菌溶血素O 432 IU/ml、类风湿因子20 IU/ml、C反应蛋白9.16 mg/L。抗核抗体阳性（1∶1 000），ENA抗体谱阴性。血小板功能检测：血小板聚集功能轻度增高。LA检测：稀释蝰蛇毒时间（DRVVT）、硅土凝固时间（SCT）的筛选比值、确认比值均升高，标准化比值在正常范围；DRVVT、SCT的混合筛选比值、混合确认比值升高，混合标准化比值升高（表1）。抗心磷脂抗体IgG 63.4 CU/ml、抗心磷脂抗体IgM 1.6 CU/ml（参考值<20 CU/ml）；抗β_2_糖蛋白1（β2GP1）抗体IgG 401.8 CU/ml、抗β2GP1抗体IgM 1.1 CU/ml（参考值<20 CU/ml）；抗磷脂丝氨酸/凝血酶原（aPS/PT）抗体IgG 823.87 U/ml（参考值0～30 U/ml）。影像学：双肺感染性病变伴出血可能性大、脾大、盆腔少量积液。

**表1:** 狼疮抗凝物检测结果

检测时间点	DRVVT检测（参考值0.8～1.2）			SCT检测（参考值0.8～1.2）		
	筛选比值	确认比值	标准化比值	筛选比值	确认比值	标准化比值
未用药（FⅡ∶C 2.7％时）	2.7	2.8	1.0	2.5	5.6	0.5
患者血浆与NPP等量混合	3.0	1.4	2.1	3.7	1.3	2.9
输注PCC 15 min后（FⅡ∶C 60％时）	2.6	1.4	1.9	4.2	1.8	2.4

**注** FⅡ∶C：凝血酶原活性；NPP：正常混合血浆；PCC：凝血酶原复合物；DRVVT：稀释蝰蛇毒时间；SCT：硅土凝固时间

患者住院期间出现晨起眼睑水肿，监测四肢血压升高（上肢153/114 mmHg、170/111 mmHg），检测24 h尿蛋白升高。考虑诊断为HLAS、系统性红斑狼疮（SLE）、高血压（继发性？），降压同时予大剂量激素冲击治疗3 d后FⅡ∶C升至17％，给予标准剂量激素治疗FⅡ∶C升至40％出院，出院1个月后复查FⅡ∶C为96％，DRVVT筛选/确认比值为1.48，提示LA存在，抗心磷脂抗体阴性，抗β2GP1抗体未检测。

## 讨论及文献复习

本例患者以出血为临床表现，实验室检查提示FⅡ∶C降低、LA阳性，因此诊断为HLAS。患者诊断过程较为复杂，提示抗磷脂抗体等物质的存在对多项凝血项目检测存在干扰，需要检验科与临床医师的密切沟通，才能达到准确诊断、及时治疗的目的。

LA是一组可以与磷脂相互作用的抗体，因其可在体外延长磷脂依赖的凝血检测时间而得名，但LA阳性患者往往表现为血栓而非出血倾向。DRVVT和SCT是目前常用的LA检测方法，2种方法原理不同，但只要有1种方法标准化比值结果阳性即可提示患者存在LA[1]。前者可以直接激活FⅩ，不受接触因子及FⅦ、FⅧ、FⅨ缺乏或抑制物的影响；后者利用接触激活物激活内源凝血途径，不受FⅦ缺乏或抑制物的影响，但2种方法均会受到LA、共同途径凝血因子（FⅡ、FⅩ、FⅤ、Fg）缺乏以及新型抗凝药（如直接凝血酶抑制剂、直接FⅩa抑制剂）的影响。临床上不建议在使用新型抗凝药物时进行LA检测，但对于本身存在共同途径凝血因子缺乏的患者，其LA检测结果解读较为复杂。

以本例DRVVT检测结果为例，入院时含有低浓度磷脂的筛选试验比值以及含有高浓度磷脂的确认试验比值均延长，后者的延长可能是由于共同途径FⅡ缺乏导致，但无法排除极强的LA等抗磷脂抗体的存在。除了常规在体外将患者血浆和等量正常混合血浆混合以补充FⅡ后再行检测外，本中心也在患者输注PCC 15 min后同时进行FⅡ∶C以及LA的检测，结果显示不管是体内还是体外，FⅡ的补充均能显著缩短确认试验比值，从而确认患者LA阳性（标准化比值>1.2），进而诊断HLAS。

HLAS在1960年被Rapaport等[2]首次报道，随后数十年，此综合征大多作为个例报道，迄今为止不足200例，我国首次于2009年由华宝来等[3]报道。研究序贯总结了1960年至2014年（90例）、2014年至2018年（18例）以及2019年至2023年（37例）报道的HLAS病例，提示HLAS的报道率呈上升趋势[4]–[6]。目前认为HLAS的核心机制主要为非中和性抗凝血酶原抗体通过加速凝血酶原清除及干扰凝血系统的正常功能，导致低凝血酶原血症发生及潜在的出血风险[7]–[8]。然而并非所有HLAS患者均可检出抗凝血酶原抗体，Ieko等[9]报道中心3年内20例HLAS患者ELISA法的抗凝血酶原抗体检出率仅为55％。因此，抗凝血酶原抗体并非诊断HLAS的必要条件。

HLAS可发生于各年龄段人群，中位年龄仅为13岁，可能与儿童肝脏网状内皮系统清除免疫复合物速度较快有关[4],[6],[10]。女性居多（56％～60％），FⅡ∶C为12％～18％[4],[6]。HLAS常伴发于自身免疫性疾病或感染，在自身免疫性疾病中最常见是SLE（70％），感染多见于病毒感染[6]。患者出血部位和程度的异质性强，从轻微的皮肤黏膜出血到危及生命的颅脑血肿均可发生，此外，其罕见性及复杂的实验室诊断，均是其极易被延误诊断的原因。

HLAS的治疗目前尚无标准，主要包括治疗原发病、控制出血和清除抗体。止血可以选用PCC、新鲜冰冻血浆等。清除抗体疗法首选糖皮质激素，静脉注射丙种免疫球蛋白以及利妥昔单抗、环磷酰胺、硫唑嘌呤等免疫抑制剂使用亦有报道[11]。与感染相关的HLAS通常持续时间短，可自行消退，总体预后良好，相反，与自身免疫性疾病或淋巴瘤相关的患者往往病程较长，治疗过程中复发性出血常见[12]–[13]。既往有患者在治疗过程中出现血栓，甚至血栓后再发致命性出血的报道[5],[14]–[16]，提示临床医师在治疗过程中应密切监测患者临床表现及凝血指标等。Bertin等[17]研究发现抗凝血酶原抗体在抗磷脂抗体综合征患者中与血栓形成有关，其定量检测可以预测血栓的复发，但在HLAS患者中能否提示血栓事件尚未可知。

## References

[1] 中国研究型医院学会血栓与止血专业委员会 (2024). 狼疮抗凝物检测与报告规范化共识[J]. 中华检验医学杂志.

[2] Rapaport SI, Ames SB, Duvall BJ (1960). A plasma coagulation defect in systemic lupus erythematosus arising from hypoprothrombinemia combined with antiprothrombinase activity[J]. Blood.

[3] 华 宝来, 范 连凯, 李 梦涛 (2010). 低凝血酶原血症-狼疮抗凝物综合征:1例报道并文献复习[J]. 血栓与止血学.

[4] Mulliez SM, De Keyser F, Verbist C (2015). Lupus anticoagulant-hypoprothrombinemia syndrome: report of two cases and review of the literature[J]. Lupus.

[5] Chen X, Nedved D, Plapp FV (2018). Fatal pulmonary embolism and pulmonary hemorrhage in lupus anticoagulant hypoprothrombinemia syndrome: a case report and review of literature[J]. Blood Coagul Fibrinolysis.

[6] Wang B, Tang N, Zhang C (2024). Lupus anticoagulant-hypoprothrombinemia syndrome: literature review and description of local case in a 3-year-old Chinese girl[J]. Semin Thromb Hemost.

[7] Bajaj SP, Rapaport SI, Fierer DS (1983). A mechanism for the hypoprothrombinemia of the acquired hypoprothrombinemia-lupus anticoagulant syndrome[J]. Blood.

[8] Edson JR, Vogt JM, Hasegawa DK (1984). Abnormal prothrombin crossed-immunoelectrophoresis in patients with lupus inhibitors[J]. Blood.

[9] Ieko M, Yoshida M, Naito S (2019). Lupus anticoagulant-hypoprothrombinemia syndrome and similar diseases: experiences at a single center in Japan[J]. Int J Hematol.

[10] Eberhard A, Sparling C, Sudbury S (1994). Hypoprothrombinemia in childhood systemic lupus erythematosus[J]. Semin Arthritis Rheum.

[11] Mazodier K, Arnaud L, Mathian A (2012). Lupus anticoagulant-hypoprothrombinemia syndrome: report of 8 cases and review of the literature[J]. Medicine (Baltimore).

[12] Sarker T, Roy S, Hollon W (2015). Lupus anticoagulant acquired hypoprothrombinemia syndrome in childhood: two distinct patterns and review of the literature[J]. Haemophilia.

[13] Meireles E, Machado F, Teles L (2020). A case report of severe bleeding due to lupus anticoagulant hypoprothrombinemia syndrome[J]. J Thromb Thrombolysis.

[14] Pilania RK, Suri D, Jindal AK (2018). Lupus anticoagulant hypoprothrombinemia syndrome associated with systemic lupus erythematosus in children: report of two cases and systematic review of the literature[J]. Rheumatol Int.

[15] Lipari A, Sorrentino S, Tamburini C (2023). Bleeding and thrombotic events in a patient with lupus anticoagulant-associated hypoprothrombinemia and antiphospholipid antibody syndromes: managing hemostasis between Scylla and Charybdis[J]. Intern Emerg Med.

[16] Nusrat S, Tewari S, Khan O (2023). Successful treatment of lupus anticoagulant hypoprothrombinemia syndrome with rituximab[J]. Thromb J.

[17] Bertin D, Beziane A, Resseguier N (2020). Interest of IgG and IgM antiprothrombin autoantibodies in the exploration of antiphospholipid syndrome: a 5-year retrospective study[J]. Rheumatology (Oxford).

